# Resilience Enhancement Online Training for Nurses (REsOluTioN): Protocol for a Pilot Randomized Controlled Trial

**DOI:** 10.2196/37015

**Published:** 2022-08-03

**Authors:** Cynthia Srikesavan, Zoe Davey, Andrea Cipriani, Catherine Henshall

**Affiliations:** 1 Oxford School of Nursing and Midwifery Faculty of Health and Life Sciences Oxford Brookes University Oxford United Kingdom; 2 Department of Psychiatry University of Oxford Oxford United Kingdom

**Keywords:** online training, nurses, resilience, mental well-being, pilot trial, COVID-19, nursing, mental health, health care staff, psychological health, online health, resilience training, health care setting

## Abstract

**Background:**

Globally, nurses are facing increased pressure to provide high-quality complex patient care within environments with scarce resources in terms of staffing, infrastructure, or financial reward. The strain and demand on the psychological health and well-being of nurses during COVID-19 has been substantial, with many experiencing burnout; as such, interventions to enhance resilience within the workplace are required. A face-to-face resilience enhancement training program for nurses that was effective in improving resilience levels was translated into a 4-week online training program, Resilience Enhancement Online Training for Nurses (REsOluTioN), to enable greater accessibility for nurses.

**Objective:**

This study aims to compare levels of resilience, psychological health, and well-being in nurses before and after the online resilience training compared to a wait list control group. It will also explore participants’ engagement with the trial and their acceptability of the online training.

**Methods:**

This is a two-arm, parallel, randomized controlled trial with a 6-week follow-up period. Up to 100 registered nonagency nurses working at a National Health Service hospital trust in South England will be recruited. Four cohorts will run, and participants will be randomized into a wait list control group or to REsOluTioN. Pre- and postonline surveys will collect study outcome measure data. In the REsOluTioN arm, data will be collected on the perceived usefulness of the online training via an online survey. Institutional and health research authority approvals have been obtained.

**Results:**

REsOluTioN will aim to empower nurses to maintain and enhance their resilience while working under challenging clinical conditions. The online training will be interactive with input from mentors, health care leaders, and peers to promote engagement and enhanced communication, and will create a forum where nurses can express their views and concerns, without hierarchical infrastructures inhibiting them. This can increase self-knowledge and learning around workplace resilience coping strategies and provide a safe space to validate feelings through mentorship and peer support. Findings will be reported in accordance with the CONSORT (Consolidated Standards of Reporting Trials) guidelines. The trial is now finished and was conducted between August 2021 and May 2022.

**Conclusions:**

The REsOluTioN trial will enable preliminary data to be gathered to indicate the online training’s effectiveness in enhancing nurses’ resilience in the workplace, with the potential for larger scale follow-up studies to identify its value to nurses working across a range of health care settings.

**Trial Registration:**

ClinicalTrials.gov NCT05074563; https://clinicaltrials.gov/ct2/show/NCT05074563

**International Registered Report Identifier (IRRID):**

DERR1-10.2196/37015

## Introduction

Worldwide, nurses are facing increased pressure to provide high-quality complex patient care to patients within environments with scarce resources in terms of staffing, infrastructure, or financial reward [1,2]. The constant strain and demand placed on nurses working in highly pressurized and often unsafe conditions, and a lack of career structure or progression means many registered nurses’ resilience levels are tested due to stress and burnout [2-4]. This stress has intensified over the last couple of years due to the COVID-19 pandemic with nurses supporting the delivery of expert patient care and rapidly responding to substantial health care challenges [1]. While nurses’ contribution to keeping the public safe and protecting the National Health Service (NHS) in the United Kingdom during COVID-19 has been widely recognized, the resulting psychological impact on health and well-being has been substantial; resilience enhancement interventions are one way to tackle this growing problem within health care services [5-8].

The NHS has been facing increased pressure due to the pandemic conditions it is being subjected to [9]. This, in addition to the chronic pressures in terms of staffing, sickness, infrastructure, and financial problems, means that nurses are facing increased pressure [2] and are experiencing burnout, as they struggle to cope with the never ceasing demands of their job roles, which are in continuous high demand [4]. The NHS Health and Wellbeing Framework [10] sets the standards for how NHS organizations should support staff to feel well, healthy, and happy at work, and advocates for the delivery of evidence-based staff health and well-being plans [10]. Resilience-building programs [11-14] are recognized as important in contributing to increased psychological health and well-being in nurses as well as aiding recruitment and retention within international health care organizations [15,16].

Recent literature has recommended that resilience-building initiatives, including mentorship programs outside of nurses’ immediate workplace settings, are incorporated into nursing education, as well as specific self-development strategies to help enhance personal resilience [15]. Resilience programs have been found to build and enhance nurses’ resilience and can also support recruitment and retention for health care workers [16]. This has been evidenced in a number of recent workplace resilience enhancement interventions within nursing [17-22].

As a result of the need for targeted resilience enhancement interventions within the nursing setting, a UK-based resilience enhancement program was developed [22,23]. A detailed description of the theoretical background of the program is available elsewhere [22]. This face-to-face program targets four core components to improve the ability of the nurses to bounce back from day-to-day problems and to remain resilient in the workplace: workplace hardiness, emotional intelligence, critical thinking, and achieving life balance and enabling spirituality. These components were informed by a 2014 integrative review [15] that reported difficult workplace situations, feelings of emptiness, and a diminishing sense of inner balance as main contributing factors for resilience in nurses. Various strategies such as reflecting on emotions, interactions and guidance from peers and mentors, and skills to develop work-life balance and toughening up are shown to be helpful in building resilience.

The program consisted of six sessions with nurses that covered topics including building hardiness, maintaining a positive outlook, achieving work-life balance, reflective and critical thinking, and enabling spirituality [22,23]. In addition, one-to-one mentor sessions were provided over a 12-week period; senior nurse leaders within the participating trust took on these mentorship roles [23]. The face-to-face program was evaluated using pre- and postsurveys and interviews with nurses. Findings showed that nurses’ resilience levels were significantly higher after the training, with reported positive impacts on personal resilience, self-awareness, confidence, well-being, and professional and team-working relationships [22]. As a result, a wider rollout of the training was proposed. However, this planned rollout was disrupted by the onset of the COVID-19 pandemic due to social-distancing measures, making the face-to-face training untenable.

Due to the restrictions imposed by COVID-19, the face-to-face training program [22,23] was adapted and transformed to online training as a means of increasing accessibility for all nurses who were no longer able to attend face-to-face training. The online training was also informed by a recent systematic review examining the effectiveness of online interventions to enhance resilience in health care professionals (C Henshall, unpublished data, 2022) and focus groups with nurses to gather information on what they felt should be key features of an online resilience training program. An online format was felt necessary due to its potential to be rolled out widely to all nurses working in health care settings due to ease of accessibility, flexible access, and a decreased need for other resources such as physical space and in-person facilitation. The online resilience training, entitled Resilience Enhancement Online Training for Nurses (REsOluTioN), is relevant in the COVID-19 climate, where consideration about how to optimize the experiences of the nursing workforce and the resulting impacts on patient care are urgently needed.

In light of the above, the randomized controlled trial described in this paper aims to pilot REsOluTioN, an online resilience training for nurses working during COVID-19. Specific study objectives are:

- To compare levels of resilience, psychological health, and well-being in nurses who complete REsOluTioN with nurses who do not (control arm)
- To explore participant engagement with the trial and acceptability of REsOluTioN
- To collect feedback on various components of REsOluTioN

## Methods

### Study Design

**Figure 1:**
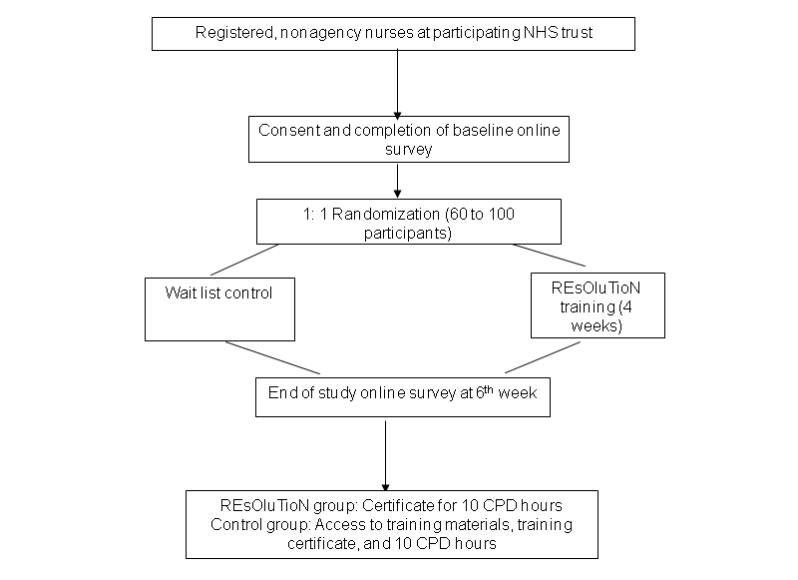
Study flowchart. NHS: National Health Service. REsOluTioN: Resilience Enhancement Online Training for Nurses. CPD: continuing professional development.

**Figure 2:**
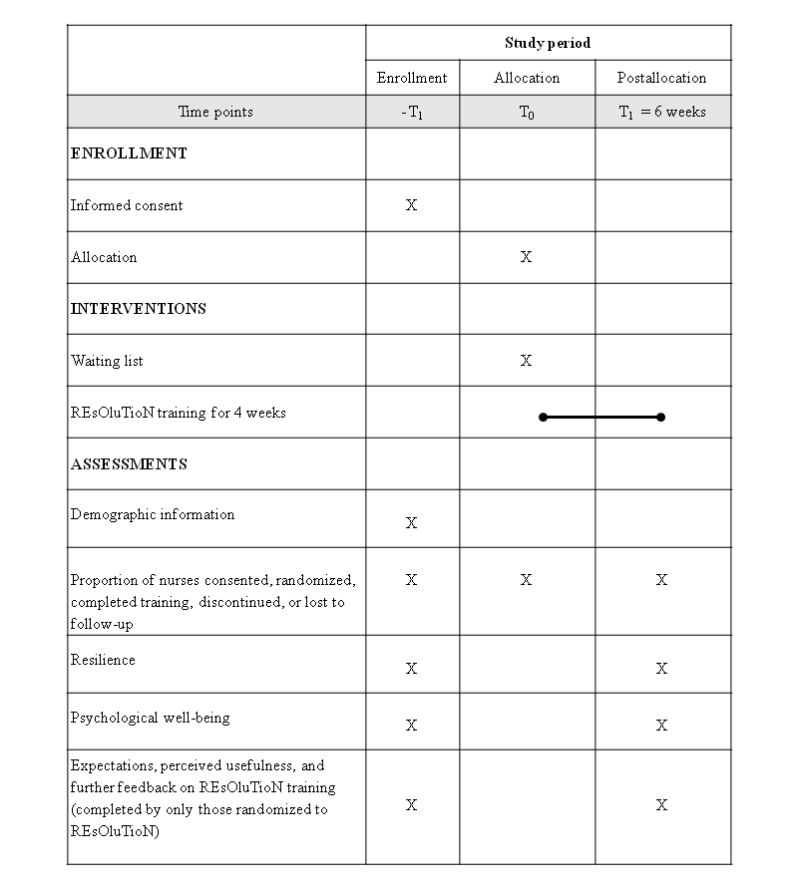
Schedule of study events and assessments. REsOluTioN: Resilience Enhancement Online Training for Nurses.

The study is a two-arm, parallel-group, individually randomized pilot trial comparing REsOluTioN versus a wait list control group. The study flowchart describing the study process is presented in Figure 1. The study schedule of events and assessments are presented in Figure 2.

### Eligibility Criteria

Any registered nonagency nurses working at any level of seniority and across any clinical setting at the participating trust will be eligible to participate. All participants must be able and willing to provide online informed consent.

### Recruitment

Nurses will be recruited through study advertisement posters and promotional videos hosted on the trust website, social media platforms, and trust premises. The researchers will attend nurse-led meetings across the trust to promote the study and will contact managers from different specialties including children, adult, and older people’s services. The researchers will also work with the hospital communications team who will include information about the study in their weekly news bulletins. The study has been endorsed by the NHS trust’s chief nurse who will be asked to share information about the trial to all nurses working in the trust, using email communications and online meeting forums. The trust’s research delivery teams will also promote the study to the clinical teams they work with. These multiple recruitment methods are deemed necessary due to the ongoing pressures facing nurses due to the pandemic, making recruitment likely to be challenging. A web link with study information included within it will be shared with people who express an interest in taking part. They will then be directed to a web page containing the online study information sheet and consent form, prior to any data collection or study procedures being undertaken. Once online consent has been confirmed, participants will be asked to complete a prestudy survey (Figure 2).

### Sample Size

Given the study objective to afford a preliminary comparison of training outcomes, as well as funding constraints and the pressures imposed by COVID-19, we propose to recruit a minimum of 60 and up to 100 participants. We aim to recruit participants over four cohorts with approximately 25 participants per cohort.

### Allocation

After providing online consent, participants will be randomized to receive either the wait list control or REsOluTioN on a 1:1 ratio. An independent research staff not involved in the design and conduct of the trial will generate a computer-generated random number sequence. Allocation concealment will be upheld using sequentially numbered opaque-sealed envelopes that will be opened sequentially only after entering the name of each participant on the envelope. A research team member who is not involved in the delivery of REsOluTioN or data analysis will implement the group allocation and contact participants to inform them of their group allocation.

### Blinding

Participants will not be blinded to their group allocation and will complete self-reported online surveys at the start of the study and after 6 weeks. It is not possible to blind the team member who undertakes the allocation assignment; however, other research team members who analyze the pre-post survey responses will be blinded to treatment allocation.

### Patient and Public Engagement

This trial evaluates an online resilience training developed for nurses and was informed by the feedback provided by nurses in the previous face-to-face training and a systematic review [22,23] (C Henshall, unpublished data, 2022).

### Interventions

#### Online Training

REsOluTioN was hosted on the Totara learning management system (version 12) by the Learning and Development (LD) information technology team of the participating NHS trust. A simple layout with clear instructions and prominent widgets to navigate the training pages will be used (Multimedia Appendices 1-3). More details about this are provided below.

##### Structure

REsOluTioN adopts a blended learning approach of asynchronous learning and synchronous sessions delivered in real time. The landing page includes a brief description about the training, module topics, library of learning materials, and a short welcome and introduction video by the study’s chief investigator.

Over a 4-week period, participants will be required to attend four facilitated online large group sessions on four module topics lasting up to 120 minutes in duration. Prior to each session, there will be 30 minutes of independent asynchronous preparatory activities. Additionally, they will attend online mentorship meetings in small groups; the mentors will all hold senior nursing positions at the participating trust. Mentor meetings will be conducted for 30 and 60 minutes twice weekly, at flexible times, over the 4-week training period.

##### Preparatory Activities

Prior to each large-group facilitated session, participants will be expected to complete some recommended readings on the module topic such as journal articles and reports, and tasks for further discussion during the sessions such as preparing responses to questions (related to module topics) such as “What does emotional intelligence mean to you and why it is important in your role?” and “What are your three main qualities of self-confidence?” The list of prereadings and tasks to be completed will be made available to participants in advance on the REsOluTioN online training page.

##### Large Group-Facilitated Sessions

Facilitated sessions will be scheduled in advance, and session dates and timings will be shared with those enrolled in each cohort. The facilitated session topics cover building hardiness and maintaining a positive outlook, intellectual flexibility and emotional intelligence, reflective and critical thinking, and achieving life balance and enabling spirituality. Experienced nurses and other multidisciplinary health care staff in senior clinical and academic positions within the local region will deliver the sessions. An outline of the content and objectives for each module is presented in Table 1.

**Table 1:** Outline of REsOluTioN training.

Components of REsOluTioN^a^	Content	Objectives
Mentorship sessions: establishing positive nurturing relationships and network	Nurturing personal relationships Relationships that encourage and motivate Crucial conversations for health care work	Focus on protective aspects of positive relationships and networks on the effects of workplace adversity
Module 1: building hardiness and maintaining a positive outlook	Defining positive outlook and personal hardiness What is known about workplace hardiness? Strategies for success: prioritizing activities in time-pressured environments, learned optimism, assertive communication An authentic life Self-esteem Self-awareness: past, present, future	Identify the elements of a positive outlook and personality hardiness related to nursing Demonstrate benefits of a positive outlook and developing hardiness for job satisfaction, health, and well-being Formulate individual strategies for improving and maintaining a positive outlook and hardiness
Module 2: IF^b^ and EI^c^	IF: definitions and characteristics Links to nursing research and resilience Dimensions of EI Emotional labor Strategies for success: expand your thinking, creative thinking, self-monitoring, expressing emotion creatively	Define principles of IF and EI Define significant existing research findings regarding IF and EI as they relate to nursing Evaluate advantages of applying elements of IF and EI to nursing practice Reflect on strategies to assist creative/critical thinking
Module 3: reflective and critical thinking	What is reflection? Making meaning of experience Moving from description to reflection Understanding reflection Therapeutic use of self Reflective awareness Reflective action	Identify importance of therapeutic use of self and reflection in expert practice Demonstrate understanding of the benefits of the reflective process to nursing practice and its underlying knowledge, influences, and motivations Define a model of reflection shown to increase critical thinking skills and develop reflexive practice Analyze individual strategies to creatively access and explore the reflection process
Module 4: achieving life balance and enabling spirituality	Work/life balance: What is balance? Why is it so hard? Envisioning work/life balance Historical, gender, and power contexts Assumptions about “juggling” Strategies for success: being comfortable doing less; adding new things to life Enabling spirituality: definition Spirituality and nursing care, health Women, spirituality, and communities	Define the importance of an awareness of work/life balance for health and well-being Demonstrate strategies for improving work/life balance Formulate historical/political background of women’s roles in caring and other work Explore some aspects of spiritually responsive nursing care available to them Explore personal perspectives on individual spirituality and its relationship to contemporary lifestyles
All modules (cross-cutting): moving forward and planning for the future	The reflective process Reflection and expert practice Strategies for success: thinking critically, reflection circuit, exhibition and participant presentations, planning for the future (goal setting)	Identify features of a resilient person and relate them to individual experiences Formulate strategies for continuation and permanency of resilient beliefs and behaviors Demonstrate an understanding of the ongoing process of resilience and the protective benefits of committing to the long-term maintenance of personal well-being

^a^REsOluTioN: Resilience Enhancement Online Training for Nurses.

^b^IF: intellectual flexibility.

^c^EI: emotional intelligence.

##### Mentor Meetings

Mentor meetings will be designed to be rewarding and valuable components of the REsOluTioN package. A mentor pool will be established by inviting around 15 experienced registered nurses from the participating trust to contribute to the study as mentors. Mentors will be registered nurses working at a Band 7 level or above. Generally, nurses working at these levels require appropriate leadership or management skills and have widespread experience of the pressures and challenges of working within the NHS. Mentors will be expected to commit to two weekly meetings with mentees for 4 weeks per cohort (mentors will be able to sign up for one or more cohorts). The mentor meetings will aim to support the mentees while they are training and to facilitate discussion relating to a wide range of topics, including but not limited to the topics covered during the facilitated sessions; the course of the discussion will be led by the mentees. The roles and expectations, issues of accountability, and key goals of the mentoring partnership will also be covered. However, this will not be a formal mentorship relationship and as such no predefined structure or mentoring plans are required.

Two separate documents with frequently asked questions to inform mentors and mentees about the structure and conduct of mentor meetings will be developed. Mentors and mentees will not be expected to maintain additional contact between the weekly meetings though they can choose to do so if they feel it would add benefit.

##### Delivery of Content

All facilitated sessions and mentor meetings will be delivered online within the parameters of confidentiality via Teams (Microsoft Corporation). Meeting links and reminders will be sent to the participants through the LD platform. Content delivery and user engagement of REsOluTioN will be in the form of text materials, illustrations, PowerPoint (Microsoft Corporation) presentations, explanatory videos, case examples, and additional reference documents such as peer-reviewed journal articles and published guidance. The facilitated sessions will also include discussions, small group work, and breakout activities. A separate YouTube account will be created for the trial, and all REsOluTioN-related videos will be made accessible only to the trial participants.

##### Time Commitment

At the end of 4 weeks, participants randomized to REsOluTioN will have completed 10 hours of structured content (2 hours of self-learning activities and 8 hours of scheduled facilitated sessions) in addition to 4-8 hours of flexible mentor meetings.

#### Control Arm

Nurses randomized to the control arm will be allocated to a wait list for 6 weeks. When they have completed the online poststudy surveys at the end of the sixth week, they will be provided access to REsOluTioN study materials.

### Outcomes

#### Participant Engagement With the Trial

Data will be collected on the number of registered nurses who expressed an interest, enrolled, were randomized, or withdrew from the study to estimate the recruitment rate and nurses’ engagement with the trial.

#### Training Outcomes

##### Acceptability of REsOluTioN

Data will be collected on the number of registered nurses who complete REsOluTioN, discontinued in between, and did not complete the posttraining survey to estimate the acceptability of REsOluTioN.

##### Resilience and Psychological Well-being

The validated Brief Resilience Scale (BRS) [24] and Warwick-Edinburgh Mental Wellbeing Scale (WEMWBS) [25] will be used to measure changes in resilience, psychological health, and well-being over time. The BRS is a brief measure of an individual’s ability to “bounce back” and has been found to have good psychometric properties compared to measures of resilience [24]. The WEMWBS has been widely used in clinical settings to measure the effects of interventions to improve well-being and can provide an indicator of resilience [25].

##### Perceived Usefulness of REsOluTioN

At baseline, the following survey data will be collected from those randomized to REsOluTioN: understanding of resilience, what they hope to gain from it, levels of perceived resilience, confidence, workplace satisfaction, and peer support.

At 6 weeks (2 weeks post training), participants’ perceived usefulness of REsOluTioN and their perceived levels of personal resilience, self-confidence, belief in own ability to provide quality patient care, relationship with work colleagues, communication skills with colleagues, outlook toward clinical practice, understanding of resilience, and feedback on REsOluTioN will be determined via an online survey.

### Data Collection Methods

Participants who expressed interest to participate in the trial will be provided a prestudy survey to provide online consent and demographic information (type of work clinical setting, banding, ethnicity, age, years of experience) before the start of each cohort and 2 weeks post cohort completion (sixth week). Surveys will be hosted on the Qualtrics online survey platform.

The surveys will contain both closed- and open-ended questions and the validated BRS [24] and WEMWBS [25]. Resilience and mental well-being scales will be completed by participants in the control and REsOluTioN arms. The closed survey questions will be completed by all participants and will take a 1 to 5 Likert-style format, with responses ranging from “not important at all” to “extremely important” or “not useful at all” and “extremely useful.” The open-ended survey questions will only be completed by REsOluTioN arm participants and will seek feedback relating to what they enjoyed the most about the training and whether any specific modules were particularly helpful.

Following completion of the postsurveys, those randomized to REsOluTioN will receive a training certificate for 10 continuing professional development (CPD) hours and those in the wait list control group will be given access to REsOluTioN materials and the 10 hours CPD training certificate.

### Data Management

Online informed consent will be obtained from all participants. All data will be stored securely in line with the university’s (study sponsor) policies for data management and storage, and in line with General Data Protection Regulation requirements.

Direct access to source data will be made only to the designated members of the study team as authorized by the chief investigator. All participant-identifiable information will be removed and anonymized using study identification codes. Electronic deidentified data will be stored and backed-up in password-protected Excel (Microsoft Corporation) spreadsheets in a secured drive accessible by the study team only. Data will be retained for 5 years after the completion of the trial.

### Statistical Methods

Demographic characteristics of the study participants will be descriptively summarized. The proportion of participants who enrolled, completed the study, provided follow-up data, or discontinued the study, and those responding to each category on the Likert-type closed questions in the survey will be reported. Pre- and postsurveys on resilience and psychological well-being will be presented as means (SDs) or medians (IQRs) depending on the normality as per Shapiro-Wilk test. The differences in resilience and psychological well-being within each arm and between arms at 6 weeks will be assessed using analysis of variance. A significance level of 0.05 will be considered. All analysis will be undertaken in SPSS statistics software, version 22.0 (IBM Corp) [26].

### Data Monitoring

The day-to-day management of the trial will be undertaken by the chief investigator and coinvestigator supported by two research associates and members of the LD team. Weekly research team meetings will be held to monitor trial conduct and progress made.

### Ethics Approval

The Faculty Research Ethics Committee at the local university approved the protocol (F.20.01.12.1, dated August 22, 2021). NHS ethical approvals were not required as patients are not being recruited to the study; however, health research authority approvals were obtained (21/HRA/1418) as well as the necessary local research and development office approvals from the participating NHS trusts. The trial protocol is registered on ClinicalTrials.gov (NCT05074563).

## Results

The trial is now completed and was conducted online via the participating NHS trust’s LD e-learning platform in England between August 2021 and May 2022.

The trial findings will be presented in accordance with the CONSORT (Consolidated Standards of Reporting Trials) reporting guidelines [27].

## Discussion

The nurse-led REsOluTioN study will aim to empower nurses to maintain and enhance their resilience while working under challenging clinical conditions. The online training delivered will be interactive, with input from mentors, health care leaders, and peers, to promote engagement and enhanced communication between nurses, their teams, and wider health care organizations (C Henshall, unpublished data, 2022). We hypothesize that the online sessions will serve as a forum where nurses can express their views and concerns, without hierarchical infrastructures inhibiting them, while increasing self-knowledge and learning around workplace resilience coping strategies and providing a safe space to validate feelings through mentorship and peer support [23]. With increasing health care pressures and a shortage of health care staff, the need to promote and support nurses’ well-being through these evidence-based interventions is vital. This is even more apparent in the current COVID-19 health care climate, where rapid and innovative approaches to patient care are constantly required; this can have a deleterious impact on health care staff due to the pressures it entails. Strategies and training programs such as REsOluTioN that promote mechanisms that enable nurses to feel better equipped to manage the daily work stressors they encounter can have short- and long-term benefits, with positive impacts on well-being, recruitment, retention, and the quality and safety of patient care [16-23].

The mentor meetings incorporated into REsOluTioN have the potential to provide nurses with a platform to vocalize any challenges, worries, and concerns they are facing in a constructive supportive environment. Effective mentee/mentor relationships and interactions with senior leaders have been found to increase resilience and to help resolve workplace challenges through empowering mentees and increasing their self-confidence, developing personal and professional growth, and supporting career progression opportunities [28-31]. Mentoring relationships can also reinforce more junior nurses’ sense of value in the workplace when they are being mentored by senior colleagues who make time to listen and respond to their problems or concerns [30]. The integration of the regular mentorship support in the REsOluTioN trial will aim to build nurses’ resilience through the promotion of professional development, self-confidence, self-worth, and problem-solving capabilities.

This study is limited to one NHS trust in England, and as a result, demographic and contextual factors may influence the value of the resource to nurses working in a range of settings and environments. As such, if the findings show that the program is effective in improving nurses’ levels of resilience, REsOluTioN will be rolled out at a national level in a larger scale randomized controlled trial design. This will enable the generalizability of the findings across a range of health care settings and contexts. In the long term, it is hoped that REsOluTioN will be a valuable resource for nurses caring for patients in challenging clinical care environments during the global pandemic and beyond.

We will publish peer-reviewed journal reports on the trial process and outcomes. Presentations will be made at national and international conferences, including the Royal College of Nursing research conference (COVID-19 permitting). We will update the Burdett Trust for Nursing (funder) with regular updates for use in newsletters and web pages. We will also collaborate and network with local research infrastructure across the region to strengthen the research profile of the project and widen the reach of our research outputs.

Trialing REsOluTioN will enable preliminary data to be gathered to indicate its effectiveness in enhancing nurses’ resilience in the workplace, with the potential for larger scale follow-up studies to identify its value to nurses working across a range of health care settings. This may lead to REsOluTioN’s widespread implementation as it is embedded within health care organizations’ overarching health and well-being strategies, with resulting improvements in the resilience of the nursing workforce and subsequent improvements in patient safety and care quality outcomes.

## Appendix

Multimedia Appendix 1 Screenshot from REsOluTioN online training: overview of training.

Multimedia Appendix 2 Screenshot from REsOluTioN online training: tabs to work through.

Multimedia Appendix 3 Screenshot from REsOluTioN online training: module 1 tasks.

## Abbreviations

BRS: Brief Resilience Scale
CONSORT: Consolidated Standards of Reporting Trials
CPD: continuing professional development
LD: Learning and Development
NHS: National Health Service
REsOluTioN: Resilience Enhancement Online Training for Nurses
WEMWBS: Warwick-Edinburgh Mental Wellbeing Scale
