# Supplementary figures and images for: Resilience Enhancement Online Training for Nurses (REsOluTioN): Protocol for a Pilot Randomized Controlled Trial
Source: JMIR Res Protoc. 2022 Aug 3;11(8):e37015. doi: 10.2196/37015 (PMC9359309; doi:10.2196/37015)

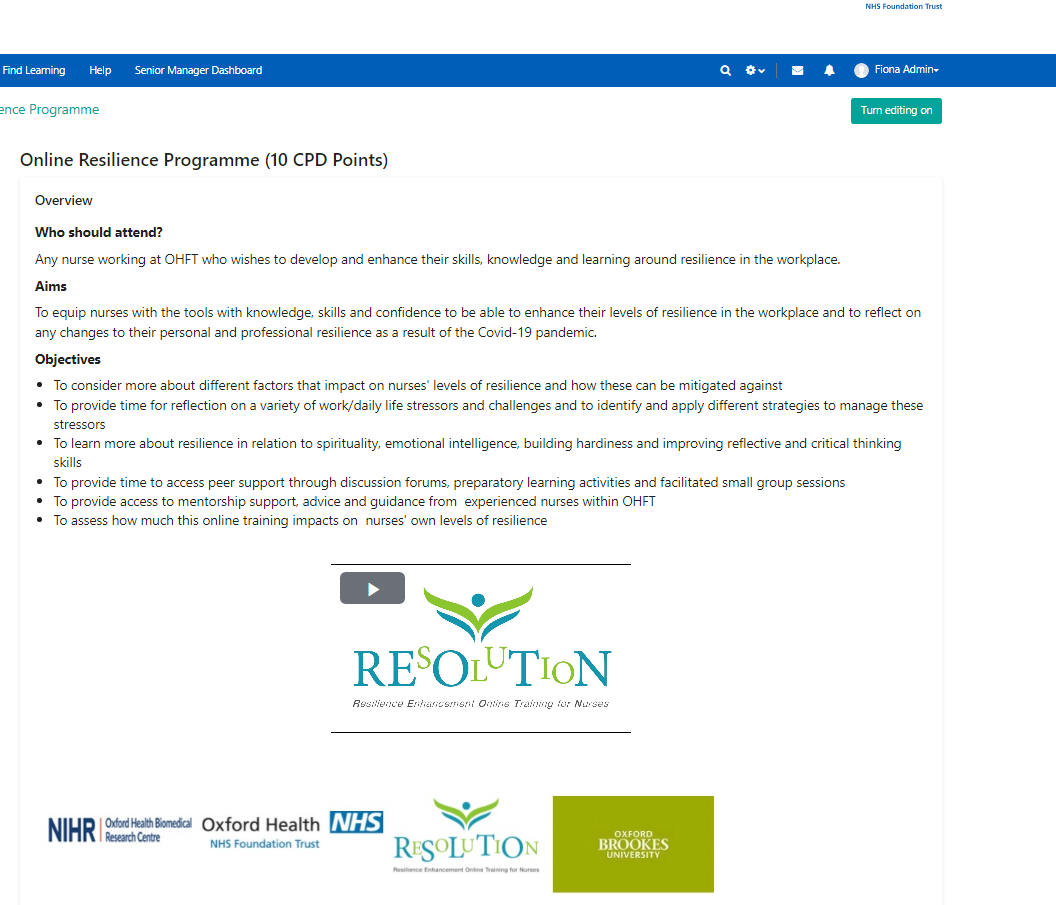

Supplement: Multimedia Appendix 1 [file resprot_v11i8e37015_app1.png]

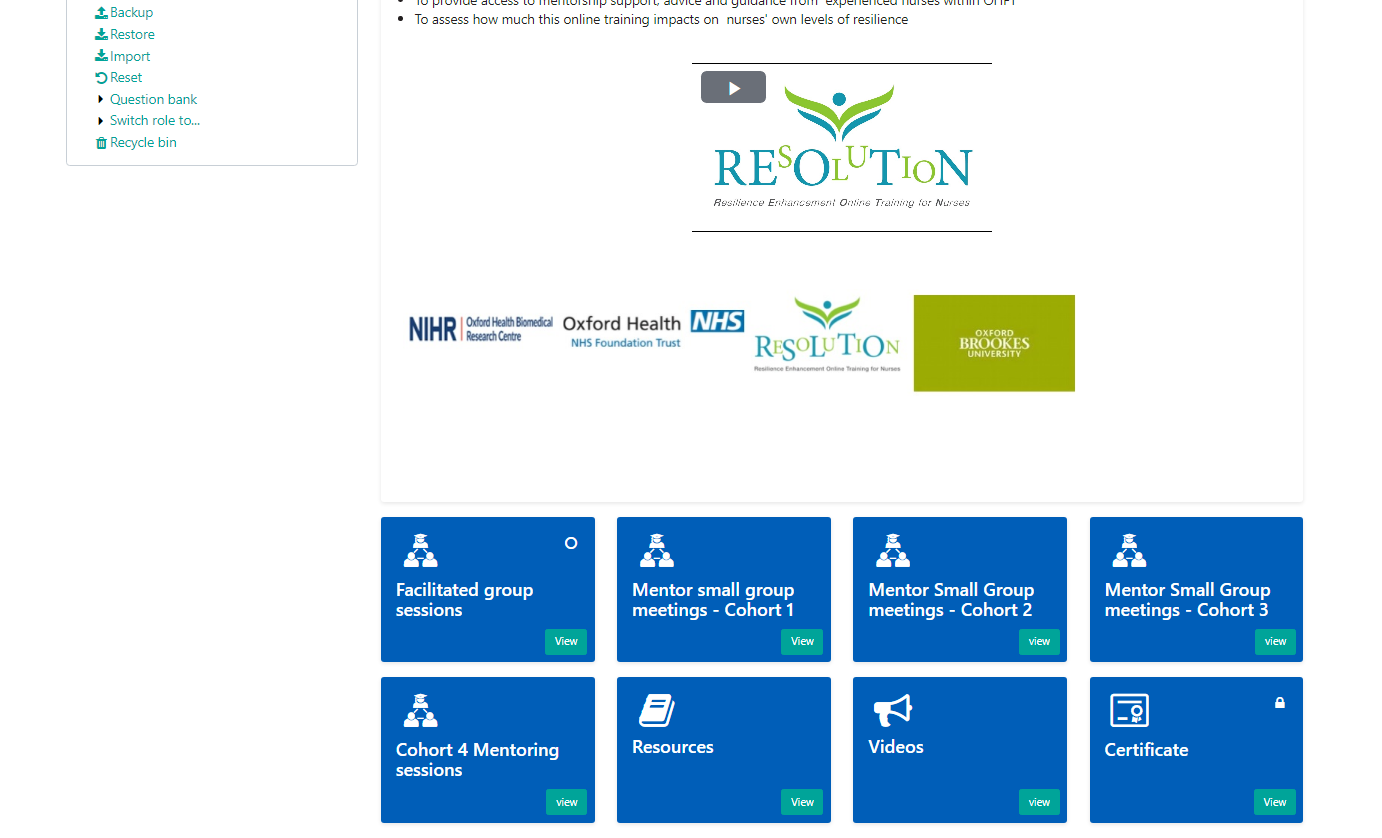

Supplement: Multimedia Appendix 2 [file resprot_v11i8e37015_app2.png]

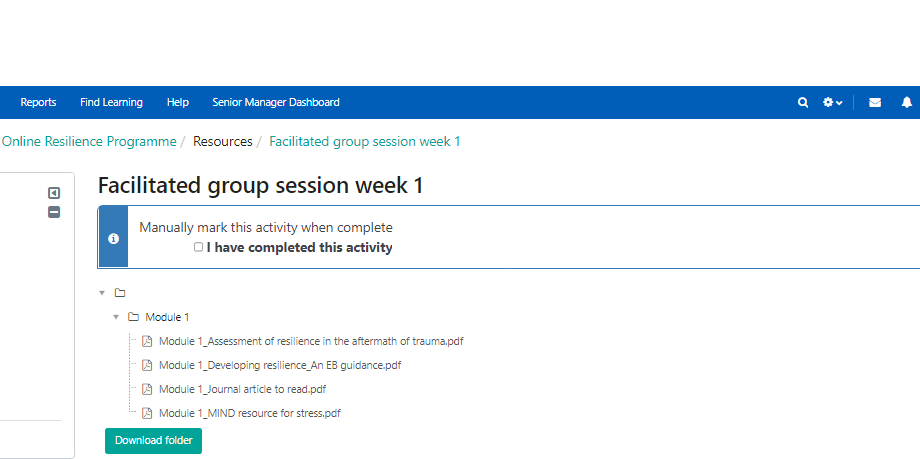

Supplement: Multimedia Appendix 3 [file resprot_v11i8e37015_app3.png]
